# The Mitotic Arrest Deficient Protein MAD2B Interacts with the Small GTPase RAN throughout the Cell Cycle

**DOI:** 10.1371/journal.pone.0007020

**Published:** 2009-09-15

**Authors:** Klaas Medendorp, Jan J. M. van Groningen, Lilian Vreede, Lisette Hetterschijt, Wilhelmina H. van den Hurk, Diederik R. H. de Bruijn, Linda Brugmans, Ad Geurts van Kessel

**Affiliations:** Department of Human Genetics, Radboud University Nijmegen Medical Centre, Nijmegen Centre for Molecular Life Sciences, Nijmegen, The Netherlands; University of Arkansas for Medical Sciences, United States of America

## Abstract

**Background:**

Previously, we identified the mitotic arrest deficient protein MAD2B (MAD2L2) as a *bona fide* interactor of the renal cell carcinoma (RCC)-associated protein PRCC. In addition, we found that fusion of PRCC with the transcription factor TFE3 in t(X;1)(p11;q21)-positive RCCs results in an impairment of this interaction and, concomitantly, an abrogation of cell cycle progression. Although MAD2B is thought to inhibit the anaphase promoting complex (APC) by binding to CDC20 and/or CDH1(FZR1), its exact role in cell cycle control still remains to be established.

**Methodology/Principal Findings:**

Using a yeast two-hybrid interaction trap we identified the small GTPase RAN, a well-known cell cycle regulator, as a novel MAD2B binding protein. Endogenous interaction was established in mammalian cells via co-localization and co-immunoprecipitation of the respective proteins. The interaction domain of RAN could be assigned to a C-terminal moiety of 60 amino acids, whereas MAD2B had to be present in its full-length conformation. The MAD2B-RAN interaction was found to persist throughout the cell cycle. During mitosis, co-localization at the spindle was observed.

**Conclusions/Significance:**

The small GTPase RAN is a novel MAD2B binding protein. This novel protein-protein interaction may play a role in (i) the control over the spindle checkpoint during mitosis and (ii) the regulation of nucleocytoplasmic trafficking during interphase.

## Introduction

The accuracy of cell cycle progression is monitored by several checkpoints in order to preserve the integrity of the DNA and to prevent the occurrence of chromosomal aneuploidy. Various components of one of these checkpoints, the mitotic spindle checkpoint, have been identified in the budding yeast *Saccharomyces cerevisiae* by studying the effects of microtubule destabilizing drugs. Subsequently, several orthologs and paralogs of the genes involved, i.e., the *BUB* and *MAD* genes, were identified in a number of species including human [1]–[6]. The high degree of sequence relatedness among these genes and its corresponding proteins suggests that they may exert conserved functional roles in cell cycle control [7]–[10]. Previously, we found that human MAD2B (MAD2L2), a protein initially identified by Cahill *et al*. (1999) [11], interacts with the renal cell carcinoma (RCC)-associated protein PRCC [12]. In addition, we found that this interaction is impaired in renal cell carcinomas carrying a t(X;1)(p11;q21) chromosome translocation and a concomitant *PRCCTFE3* gene fusion. MAD2B can bind indirectly to the anaphase promoting complex APC. The APC, in turn, can be activated through cell cycle-dependent associations with regulatory components such as CDC20 or CDH1 (FZR1) [13]. MAD2B is thought to inhibit APC^CDH1^ and APC^CDC20^ by binding to CDH1 and CDC20, respectively [3], [4]. The closely related cell cycle checkpoint protein MAD2 (MAD2L1) exerts a similar inhibitory effect through binding to CDC20 [14], [15]. In order to further delineate the putative role of MAD2B in cell cycle control, we performed a protein-protein interaction screen using MAD2B as a bait. By doing so, we identified the small GTPase RAN, a well-known cell cycle regulator [16], as a novel and *bona fide* MAD2B-interacting protein. Based on our data and those reported by others, we propose that this newly identified protein-protein interaction may play a role in (i) the control over the spindle checkpoint during mitosis and (ii) the regulation of nucleocytoplasmic trafficking during interphase.

## Materials and Methods

### Yeast two-hybrid assays

Yeast two-hybrid assays were performed essentially as described before using a hybriZAP human testis cDNA library containing approximately 4×10^6^ independent cDNA clones with an average insert size of ∼1 kb [12], [17]. Of these, 1×10^6^ clones were amplified once and 1×10^9^ of the resulting plaque forming units were mass-excised according to the manufacturer's instructions to generate an interaction cDNA library in pAD-GAL4. The yeast strain used for the interaction assays was pJ69-4A (a kind gift from Philip James). Positive interactions in this yeast strain can be selected for by adenine and histidine auxotrophy, next to β-galactosidase activity. The pJ69-4A strain was co-transfected with bait plasmid pBDT3C, carrying the human MAD2B coding sequence (AL031731), and the testis cDNA library using the Yeastmaker transformation kit (Clontech). To select double transfectants, containing a pBD and a pAD vector, the transfected yeast cells were plated on synthetic defined (SD) medium lacking leucine and tryptophan (-LW). All resulting colonies were recovered from these plates, titrated, and re-plated on SD medium lacking histidine, adenine, leucine and tryptophane (-HALW) to reselect yeast clones expressing MAD2B interacting proteins. Colonies were allowed to grow for at least 5 days at 30°C after which replicate filters were lifted and tested for β-galactosidase activity according to the manufacturer's instructions (LacZ filter lift assay; Stratagene). The cDNA inserts of positive clones were isolated by direct PCR on yeast colonies with two pAD-GAL4 specific oligonucleotides, pAD-for (CTGTCACCTGGTTGGACGGACCAA) and pGAD-rev (GTGAACTTGCGGGGTTTTTCAG). All resulting PCR products were sequenced using the same oligonucleotides, and the pAD plasmids of these yeast clones were rescued in DH5α bacteria. Finally, the interacting cDNA clones were retransfected together with control plasmids as reported before [17].

### Cloning and sequencing procedures

All cloning procedures were essentially as described before [17], [18]. Sequence analyses were performed at the DNA sequence facility of the Radboud University Nijmegen Medical Centre using a 3730 DNA Analyzer (Applied Biosystems). DNA and protein databases were searched using the BLAST or BLAT search algorithms at the NCBI or UCSC, respectively. Deletion constructs of human MAD2B were generated using primers dispersed at intervals of 150 nt throughout the cDNA sequence. The 5′-end of the forward primers were located at amino acid positions 1, 51, 101 and 151, respectively, according to NP_006332. The reverse primers were located at positions 211, 161, 111 and 61. The resulting PCR products were cloned into pGEM-T (Promega), sequence-verified, and subcloned into the correct pBDT3C and pGADT3C vectors. Full-length human MAD2B was cloned in pDEST53 (Invitrogen), pECFP (Clontech), pDEST303 (Invitrogen), pSG8-VSV [12] and pDEST306 (Invitrogen), yielding MAD2B-GFP, MAD2B-CFP, HA-MAD2B, VSV-MAD2B and FLAG-MAD2B fusion proteins, respectively. Full-length PRCC was cloned in pCMV-dsRed (Clontech), yielding a dsRed-tagged fusion protein (PRCC-dsRed). The full length RAN coding sequence was cloned into pDEST15 (Invitrogen) and pEYFP (Clontech), yielding GST-RAN and RAN-YFP fusion proteins, respectively.

### Cell culture

COS-1, U2OS and HEK293 cells were cultured in DMEM (Invitrogen) supplemented with 10% FCS, penicillin (100 U/ml) and streptomycin (100 µg/ml) at 37°C and 7,5% CO_2_. For the cellular co-localization studies, cells were transiently transfected using an Amaxa Nucleofector according to the instructions of the manufacturer (Lonza). For localization of the proteins, cells were fixed in 3.7% paraformaldehyde/PBS, pH 7.4 for 30 minutes at room temperature, permeabilized in PBS/0.2% Triton, and embedded in Vectashield with DAPI (Vector Labs.). Subsequently, the cells were analyzed by immunofluorescence and/or confocal laser scanning microscopy as reported before [17]. Cells were synchronized in M- or S-phase through a 16 h incubation with 250 ng/ml nocodazole or 2.5 mM hydroxyurea, respectively. Subsequently, the drugs were removed and the cells were maintained in culture for various time periods as indicated. The resulting cell cycle distribution of the cells was determined by Fluorescence Assisted Cell Sorting (FACS) analysis as described before [18], [19] using 10^6^ cells/ml supplemented with 40 µg/ml Propidium Iodide.

### GST pull-down and co-immunoprecipitation assays

GST-RAN was transfected into BL21-DE3 cells (Promega) and purified as described before [20]. For the GST pull-down assays, 50 µl of GST-RAN was combined with 100 µl of lysates from U2OS cells expressing HA-MAD2B. In addition, 50 µl glutathione Sepharose 4B slurry (Amersham Biosciences) was used. A protease inhibitor cocktail (Roche) was added during all wash steps. Before use, the sepharose beads were washed twice with TBS (25 mM Tris pH 7.4 and 150 mM NaCl), followed by a 2 h incubation of the GST-RAN (and GST alone as a control) with the beads. After this incubation, the GST-coupled beads were washed with TBSTD buffer (25 mM Tris pH 7.4, 150 mM NaCl, 1.0% Triton X-100 and 2 mM DTT), and incubated overnight at 4°C with lysates of U2OS cells expressing HA-MAD2B (see above). After five additional washes with lysis buffer (50 mM Tris-HCl pH 7.5, 150 mM NaCl and 0.5% Triton X-100) and washing buffer (50 mM Tris-HCl pH 7.5, 100 mM NaCl, 2 mM MgCl_2_, 2 mM CaCl_2_, 1% Triton X-100 and 2 mM DTT) the samples were denatured and loading onto 4–12% NuPAGE polyacrylamide gels (Invitrogen). After electrophoreses the gels were blotted onto nitrocellulose membranes (Protran, Schleicher and Schuell). The resulting blots were blocked in PBS with 5% non fat dry milk and incubated with the anti-MAD2B antibody for 1 h at room temperature and, subsequently, with either a peroxidase conjugated secondary antibody (Zymed), or a fluorescent conjugated secondary antibody (Molecular Probes). Peroxidase signals were visualized using auto-radiographic exposure to Kodak X-Omat films, while fluorescent signals were scanned and analyzed with the Odyssey system and its associated software (Li-Cor). For the immunoprecipitation assays, cells were washed three times in ice-cold PBS and scraped from the tissue culture dishes. Total cell lysates were prepared in 1 ml lysis buffer (PBS,10% glycerol, 0.1% NP40, protease inhibitors), kept on ice for 10 min, while vortexed gently at 2 min intervals, and centrifuged for 30 min at 38.500 rpm at 4°C. The supernatants were pre-cleared in 100 µl of protein A/G PLUS Sepharose (50% slurry in lysis buffer; Santa Cruz) for 1 h at 4°C and subsequently centrifuged for 3 min at 2500 rpm at 4°C. Immunoprecipitations were performed with these pre-cleared supernatants by adding 1–5 µg primary antibody, followed by a 1 h rotation at 4°C. Subsequently, 25 µl protein A/G PLUS Sepharose was added to the immune complexes for 1 h at 4°C. The sepharose-bound immune complexes were collected by centrifugation (3 min; 2500 rpm; 4°C). The resulting pellets were washed five times with lysis buffer before antigens were released by heating at 95°C for 5 min. Western blotting was performed as described above.

### Antibodies

A polyclonal rabbit anti-MAD2B antibody was used as described before [12]. Monoclonal mouse anti-RAN (610340; BD Biosciences), monoclonal mouse anti-H-RAS (sc-29; Santa Cruz Biotechnology Inc.) and monoclonal mouse anti-FLAG (F1804; Sigma) antibodies were used according to the instructions of the manufacturers. The anti-γ-tubulin antibody used was kindly provided by Bé Wieringa (Nijmegen, The Netherlands).

## Results

### Identification of the small GTPase RAN as a MAD2B binding protein

To search for novel MAD2B interactors, we used a testis cDNA library for yeast-two-hybrid screening as described before (see Materials and methods). Transfection of the MAD2B-bait alone in yeast cells did not yield any growth on histidine-deficient plates, as expected. Co-transfection of the bait with the human testis cDNA interaction library resulted in 92 colonies that grew on fully selective medium. Of these, 69 were selected for further analysis. Sequencing revealed that two of these yeast colonies contained pAD plasmids encoding the C-terminal 60 amino acids (aa 157–216) of the small GTPase RAN. Co-transfection of these pAD-RAN plasmids with standard control plasmids encoding lamin-B and p53 turned out to be negative. However, yeast cells co-transfected with the MAD2B bait and the pAD-RAN plasmids were able to grow on fully selective medium and turned blue in a β-galactosidase assay, thereby validating the yeast two-hybrid interactions observed.

**Figure 1:**
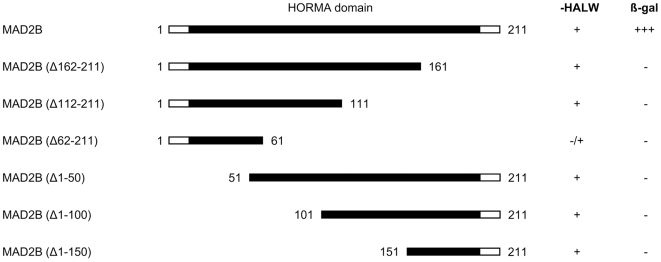
Yeast two-hybrid analysis of full length RAN with MAD2B deletion constructs. Schematic representation of the MAD2B full length protein and deletion constructs (numbers correspond to amino acid positions) used in the assay and the capacity of the respective co-transfected yeast cells to grow on selective medium (-HALW). The presence of β-galactosidase activity (β-gal) as measured in filter lift assays is indicated by +.

In order to establish whether MAD2B is also capable of interacting with full-length RAN, we subsequently introduced its corresponding sequence into the yeast two-hybrid system. Conversely, in order to identify the region of MAD2B responsible for the interaction with RAN, several deletion constructs were generated lacking consecutive stretches of 50 amino acids at either the C- or N-terminal regions of the coding sequence (Fig. 1). The resulting MAD2B (deletion) constructs were transfected into yeast cells together with full-length RAN and all transfectants were tested for their ability to grow on fully selective medium and for β-galactosidase activity. Yeast cells, transfected with full-length RAN and full-length MAD2B (amino acids 1–211) grew well and showed a strong β-galactosidase activity and, thus, a strong interaction. Transfection of MAD2B deletion constructs with full-length RAN, however, resulted in a complete abrogation of yeast growth and β-galactosidase activity (Fig. 1) indicating that full length MAD2B is required for the interaction with RAN. Since the MAD2B protein almost entirely consists of the HORMA domain [21], and any deletion which affects this domain results in abrogation of the interaction, it is likely that the whole domain is required for the interaction between MAD2B and RAN. In contrast, the C-terminal 60 amino acids of RAN appear to be sufficient for the interaction with MAD2B.

### Endogenous MAD2B and RAN interact physically

**Figure 2:**
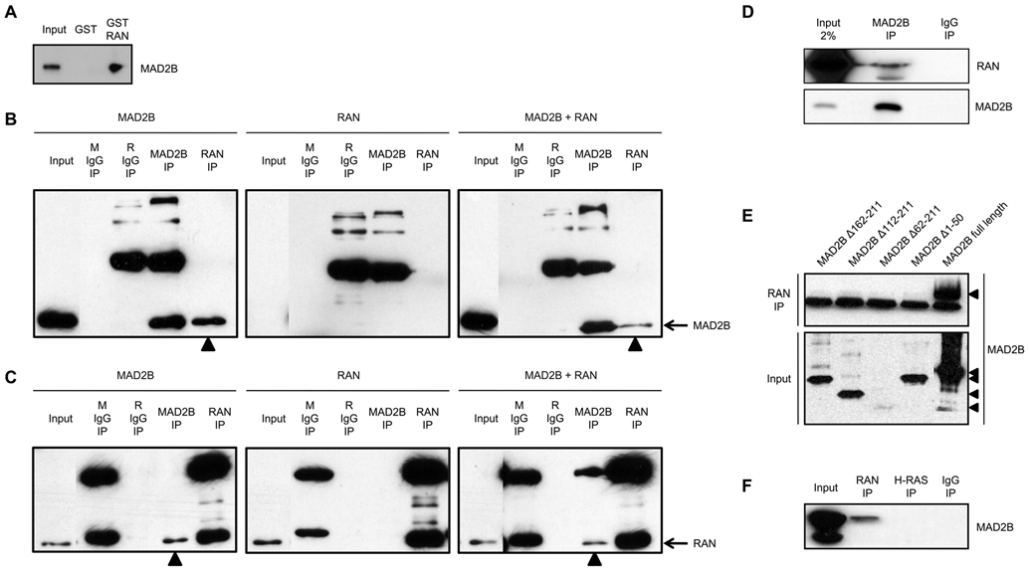
Co-immunoprecipitation of MAD2B and RAN proteins. (A) U2OS cells expressing HA-MAD2B protein were lysed and probed for interaction with either GST-RAN or GST alone. Specific interaction between MAD2B and RAN was detected by western blot analysis using an anti-MAD2B antibody. (B, C) COS-1 cells were transiently transfected with MAD2B, RAN or both as indicated on top. Immunoprecipitations were performed with pre-mouse serum (M IgG), pre-rabbit serum (R IgG), anti-MAD2B and anti-RAN antibodies as indicated. Western blot analyses were performed with (B) an anti-MAD2B antibody, and (C) an anti-RAN-antibody. The positions of the immunoreactive MAD2B and RAN proteins are marked on the right. The (co-)immunoprecipitated MAD2B (B) and RAN (C) proteins are marked with arrowheads. The background bands represent immunoglobulin heavy and light chains, respectively. (D) Immunoprecipitations were carried out with an anti-MAD2B antibody and a control rabbit antibody (IgG) on HeLa cell lysates as indicated. Western blots analyses were performed with an anti-RAN (upper panel) and an anti-MAD2B (lower panel) antibody, respectively. (E) COS-1 cells were transiently transfected with FLAG-MAD2B protein and FLAG-tagged deletion constructs of MAD2B as indicated (see also Fig. 1). Upper panel: immunoprecipitations with an anti-RAN antibody, lower panel: input lysates. The background bands in the upper panel represent immunoglobulin light chains. Western blot analyses were performed with an anti-FLAG antibody. (F) COS-1 cells were transiently transfected with MAD2B and immunoprecipitations were performed with anti-RAN and anti-H-RAS antibodies, respectively. A pre-mouse serum (IgG) was included as a negative control. Western blot analysis was performed with an anti-MAD2B antibody.

To investigate whether the MAD2B protein interacts directly with RAN, glutathione S-transferase (GST) pull-down analyses were performed, using GST-RAN fusion proteins produced in *E. Coli*. Full-length HA-tagged MAD2B (HA-MAD2B) proteins were obtained by transfecting U2OS cells, leading to the generation of a SDS-PAGE protein band of expected size, 28–29 kDa (Fig. 2A; Input). After performing GST pull-down assays using the GST-RAN fusion protein and GST alone, we observed that the MAD2B protein co-precipitated with the GST-RAN fusion protein (Fig. 2A), but not with the GST protein, thus confirming a direct interaction between MAD2B and RAN.

In order to assess the MAD2B-RAN interaction in mammalian cells, we transiently transfected COS1 cells with VSV-MAD2B and/or RAN-YFP constructs. Subsequently, immunoprecipitations were performed on single and double transfectants, using anti-MAD2B and anti-RAN antibodies to precipitate the respective proteins (Fig. 2B,C). The resulting immunoprecipitation products were analyzed using western blotting in conjunction with either anti-MAD2B (Fig. 2B) or anti-RAN (Fig. 2C) antibodies. Total lysates were loaded as controls (Fig. 2B,C; Input). In single MAD2B and double MAD2B/RAN transfectants, MAD2B was readily detected as a ∼28 kDa protein (including the VSV-tag; Fig. 2B) which was not present in single RAN transfectants. Conversely, in single RAN and double MAD2B/RAN transfectants, as well as in untransfected cells, RAN could readily be detected as a ∼21 kDa protein (Fig. 2C), which corresponds to the size of the endogenous protein. Based on this latter observation we conclude that the anti-RAN antibody used does not recognize RAN-YFP protein on western blot. Subsequently, protein extracts from cells transfected with MAD2B were subjected to immunoprecipitation with the anti-RAN antibody. Analysis of these precipitates by western blotting with the MAD2B antibody revealed co-immunoprecipitation of MAD2B with RAN, but not with a pre-immune control (Fig. 2B). This physical interaction between exogenous MAD2B and endogenous RAN was also confirmed *vice versa*, i.e., after immunoprecipitation with the anti-MAD2B antibody endogenous RAN could readily be detected (Fig. 2C). The variations observed in band intensities are due to variations in the respective transfection efficiencies. In order to exclude cell-type specific effects, these immunoprecipitations were replicated in HEK293 cells in the same fashion as outlined above. Again, we observed co-immunoprecipitation of exogenous MAD2B and endogenous RAN (data not shown).

Finally, we performed anti-MAD2B immunoprecipitations with protein extracts from non-transfected HeLa cells. Western blotting using the anti-RAN antibody, revealed the presence of the endogenous RAN protein both in the input and the anti-MAD2B immunoprecipitate, but not in the mock (IgG) immunoprecipitate (Figure 2D), thus supporting a completely endogenous interaction between MAD2B and RAN. Taken together, these combined immunoprecipitation analyses confirm the presence of an *in vivo* physical interaction between these two proteins in mammalian cells.

Immunoprecipitation of several (FLAG-tagged) MAD2B deletion fragments that were also used in our yeast two-hybrid screens failed to pull down RAN (Fig. 2E), indicating that, in full conformity with the yeast two-hybrid data, also in mammalian cells full-length MAD2B is required for the interaction with RAN. In order to assess the specificity of the MAD2B-RAN interaction, we included RAN family member H-RAS as a negative control. Again co-immunoprecipitation of MAD2B with RAN was readily detected, whereas no co-immunoprecipitation of MAD2B with H-RAS was observed (Fig. 2F), thus confirming that the physical interaction observed between MAD2B and RAN is specific.

### MAD2B and RAN co-localize within mammalian cells

**Figure 3:**
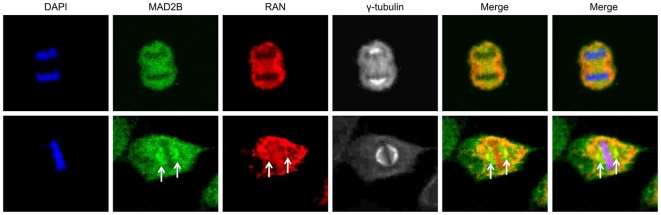
Sub-cellular (co-)localization of MAD2B and RAN. Endogenous MAD2B, RAN and γ-tubulin (mitotic spindle) are shown in green, red and white, respectively. DAPI staining (blue) was used to mark nuclei and chromosomes. The overlay of the different signals (Merge) reveals a near perfect co-localization of the respective proteins at the mitotic spindle (arrows). Images were captured using confocal laser scanning microscopy.

To assess whether the observed interaction between MAD2B and RAN is operative in mammalian cells, we determined the sub-cellular localization of both proteins in U2OS cells. To this end, an expression construct carrying the full coding sequence of GFP-tagged MAD2B was transiently transfected into U2OS cells, after which the sub-cellular localizations of MAD2B and (endogenous) RAN were assayed by fluorescence microscopy. By doing so, we observed a (near) perfect co-localization of both proteins during interphase (Figure S1A). In order to substantiate these results, we performed complete endogenous co-localization assays in U2OS cells in conjunction with confocal laser scanning microscopy. The results obtained are in full conformity with the above notion that MAD2B and RAN co-localize in interphase cells. In addition, we found a perfect co-localization at the spindle, i.e., γ-tubulin, during mitosis (Fig. 3). In order to further specify this localization at the mitotic spindle, we were able to exclude that endogenous RAN co-localizes with histone-2B (Figure S1B), thus indicating that RAN is not associated with chromatin. Based on these combined results, we conclude that MAD2B and RAN share similar sub-cellular localizations in mammalian cells, both during interphase and mitosis.

### The MAD2B-RAN interaction persists throughout the cell cycle

**Figure 4:**
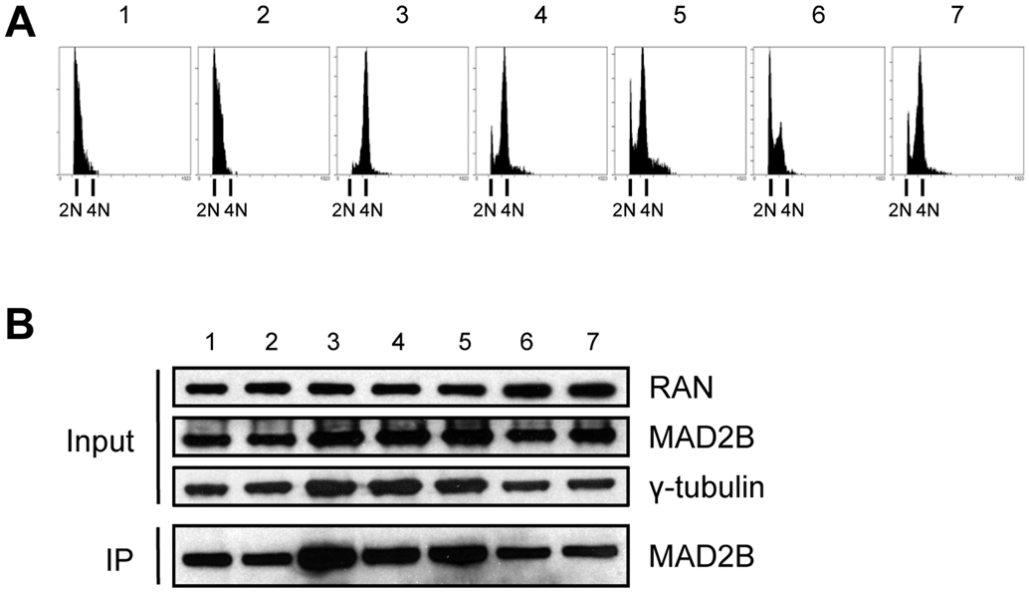
MAD2B and RAN interact throughout the cell cycle. COS-1 cells were transiently transfected with MAD2B, blocked at different phases in the cell cycle and, subsequently, released from these cell cycle blocks during different time intervals. (1) cells isolated from a confluent culture, (2) cells isolated 3 h after 1∶2 splitting of a confluent culture, (3) cells blocked with nocodazole, (4) cells blocked with nocodazole and released for 4 h, (5) cells blocked with nocodazole and released for 8 h, (6) cells blocked with hydroxyurea, and (7) cells blocked with hydroxyurea and released for 6 h. (A) FACS profiles confirming the presence of the cells in the different phases of the cell cycle are shown. (B) Western blot analysis of total lysates with anti-RAN, anti-MAD2B and anti-γ-tubulin antibodies (Input) and after anti-RAN immunoprecipitation of the respective lysates (1–7) using an anti-MAD2B antibody (IP).

Since MAD2B and RAN have been proposed to regulate various cell cycle processes [3], [4], [22], we decided to assess whether the observed interactions between these two proteins may relate to different phases of the cell cycle. To this end, COS-1 cells were transfected with VSV-MAD2B and, subsequently, aliquots of these transfected cells were synchronized by adding either nocodazole (G2/M phase block) or hydroxyurea (S phase block). Subsequently, the synchronized cells were released from drug-treatment during several time intervals as indicated (Fig. 4). To confirm the efficiency of synchronization, cells were fixed and analyzed by FACS at all time intervals. After harvesting cells from confluent cultures, and cultures that were split and allowed to grow for 3 h, most cells were found to be in the G0/G1 phase of the cell cycle, as expected (Fig. 4A, panel 1 and 2, respectively; 2N). After nocodazole treatment, most cells were arrested in the G2/M phase of the cell cycle, again as expected (Fig. 4A, panel 3; 4N), whereas subsequent release from this block for 4 and 8 h resulted in passage of the cells through mitosis and re-entry into the G0/G1 phase of the cell cycle (Fig. 4A; panel 4 and 5, respectively). Also as expected, treatment of the cells with the DNA replication inhibitor hydroxyurea provoked a block in the S phase, whereas after a release of this block for 6 h most of the cells were in the G2/M phase of the cell cycle (Fig. 4A; panel 6 and 7, respectively).

Simultaneous to these analyses, cell lysates were prepared from the above described samples (1–7) and assayed for the presence of MAD2B and RAN proteins using western blotting. As a loading control anti-γ-tubulin was included. The same lysates were also used for co-immunoprecipitation experiments in order to assess the putative state of interaction between MAD2B and RAN during the different phases of the cell cycle. In all lysates tested endogenous RAN and exogenous MAD2B were found to be present (Fig. 4B; Input). The levels of RAN and MAD2B protein showed no overt differences between the (sub)confluent, nocodazole and/or hydroxyurea treated cells (Fig. 4B; Input). Immunoprecipitations with anti-RAN antibody followed by western blotting and detection with anti-MAD2B antibody revealed co-immunoprecipitation during all phases of the cell cycle tested (Fig. 4B; IP). From these data we conclude that the MAD2B-RAN interaction observed persists throughout the cell cycle.

### MAD2B interacts with either RAN or PRCC

**Figure 5:**
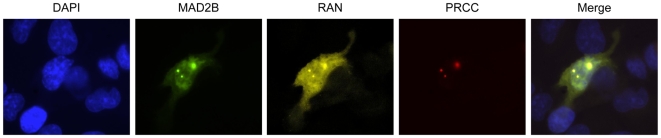
Sub-cellular (co-)localization of MAD2B, RAN and PRCC in U2OS cells. U2OS cells were transiently (co-)transfected with MAD2B-CFP (green), PRCC-dsRed (red) and/or RAN-YFP (yellow) expression constructs. The overlay of the different signals (Merge) reveals a partial co-localization of the respective proteins. Images were captured using fluorescence microscopy.

Previously, we found that MAD2B can interact with the renal cell carcinoma associated protein PRCC [12]. To assess whether PRCC may act as an integral component of the MAD2B-RAN complex, U2OS cells were transiently (co-)transfected with MAD2B-CFP, PRCC-dsRed and/or RAN-YFP expression constructs. Subsequently, the transfected cells were assayed for the sub-cellular localization of the respective proteins. By doing so, we found that single PRCC transfectants showed a nuclear staining pattern as reported before [12]. Double MAD2B and PRCC transfectants showed a nuclear co-localization, again in accordance with our previous observations [12]. Triple MAD2B, PRCC and RAN transfectants showed a partial co-localization of MAD2B, RAN and PRCC (Fig. 5). As a control, to again exclude chromatin association, we tested whether endogenous PRCC co-localizes with histone-2B. This was not found (Figure S2). Taken together, we conclude that MAD2B, RAN and PRCC share similar sub-cellular compartments. The partial co-localization observed between these three proteins indicates that the interactions of MAD2B with either RAN and/or PRCC may be dynamic in nature. To test whether PRCC and RAN can interact directly, we performed co-immunoprecipitation analyses. By doing so, no co-immunoprecipitation between PRCC and RAN was observed, thus excluding a direct interaction between these two proteins (data not shown).

## Discussion

Using a yeast two-hybrid interaction trap we identified the small GTPase RAN as a novel MAD2B interacting protein. An endogenous interaction of these proteins was confirmed in mammalian cells using several *in vitro* and *in vivo* assays. Co-expression of MAD2B and RAN in COS-1 cells revealed a near perfect co-localization in both during interphase and mitotis. Using confocal laser scanning microscopy, we found that the respective endogenous proteins exhibited very similar sub-cellular co-localization patterns, in particular at the mitotic spindle. A concomitant physical interaction was found to be present throughout the different phases of the cell cycle tested.

The interaction domain of RAN could be assigned to its C-terminal moiety. A previous analysis of the 3-dimensional structure of RAN has revealed that, when RAN is complexed with importin β-related proteins, its C-terminal moiety is exposed at the outside of the molecule [23]–[25]. Such an exposure may render RAN accessible for interactions with other proteins such as MAD2B. On the other hand, full length MAD2B protein appeared to be required for the interaction with RAN, as observed in both our co-immunoprecipitation and yeast two-hybrid analyses. This requirement may be related to a proper folding of the protein and/or a proper exposure of its interaction interface(s). Alternatively, the formation of multimeric MAD2B configurations, a feature that has been documented extensively for the various protein-protein interactions exhibited by its closely related homolog MAD2 [26], [27], may be essential for the MAD2B-RAN interaction.

There is ample evidence indicating that RAN is at the regulatory heart of several processes governing the proper execution of mitosis [28], [29]. The mechanisms through which RAN controls these processes, however, are not yet fully understood. Our current results suggest that MAD2B, through its interaction with RAN, may also be involved in these processes. A suggestion for such a role comes from the observation that ribonucleoprotein complexes, which are transported across the nuclear membrane, are required for a correct mitotic spindle assembly [30]. Next to RAN (this report) and PRCC [12], trichosanthin (TCS) [31], HCCA2 [32], Elk-1 [33], IpaB [34], TCF4 [35] and adenovirus death protein (ADP) [36] have been identified as MAD2B binding proteins. ADP is a membrane spanning protein which mediates adenovirus-induced cell lysis. Since efficient nucleocytoplasmic transport of viral RNAs is essential for cell lysis, and since overexpression of MAD2B results in the inhibition thereof [36], MAD2B may also be linked to ribonucleoprotein transport across the nuclear membrane and, thus, mitotic spindle assembly.

Another indication that suggests a more general RAN-associated function in cell cycle control comes from the observation that RAN may exhibit a dual role, depending on the RAN binding protein RANBP1. The RAN-RANBP1 complex has been implicated in cell cycle progression and RNA export, while RAN alone has been implicated in nuclear protein import. By competing with RANBP1 for binding to RAN [31], [36], MAD2B may affect the export of RNA and, thus, the targeting of ribonucleoprotein complexes. Such a role would also be compatible with the here observed interaction between MAD2B and RAN and the perseverance of this interaction throughout the different phases of the cell cycle.

Proteolysis and spatial control are two key mechanisms that regulate critical events during the cell cycle. An important mediator of these cell cycle-associated processes is the anaphase promoting complex (APC) [13]. Previously, it was found that the RAN-dependent complex Rae1/Nup98, which shares both sequence and structural homologies to several BUB checkpoint factors, interacts with the CDH1 regulatory component of the APC. In addition, it was found that elevated levels of Rae1/Nup98 prevent APC^CDH1^-dependent proteolysis of securin and, thereby, exit from mitosis. Conversely, dissociation of Rae1/Nup98 from APC^CDH1^ was suggested to contribute to mitotic progression [37]. Our results point at a similar RAN-dependent mitotic control function via its interaction with the CDH1-binding protein MAD2B [3], [4]. The APC^CDH1^ complex is also crucial for maintaining cells in the G1 phase of the cell cycle in order to prevent premature progression into the S phase. Nuclear export of CDH1 relies on RAN GTPase and results in the inhibition of APC activity [38]. Since MAD2B can interact with both CDH1 and RAN, we assume that this protein may also affect nucleocytoplasmic transport of CDH1 and, consequently, the activity of the APC^CDH1^ complex during the G1 phase of the cell cycle. Thus, through its association with RAN, MAD2B may be involved in controlling the transport of CDH1 across the nuclear membrane and, by doing so, the transition from the G1 to the S phase of the cell cycle as well.

Since we have found that exogenous co-expression of MAD2B and PRCC results in translocation of MAD2B from the cytoplasm to the nucleus where it co-localizes with PRCC, we previously suggested that MAD2B may travel across the nuclear membrane when bound to PRCC [12]. This suggestion is in line with our subsequent observation that MAD2B contains a potential nuclear localization signal [6]. Alternatively, however, MAD2B may travel across the nuclear membrane when bound to RAN, a notion that would be in agreement with our current PRCC co-localization assays and our suggestion that the respective MAD2B-PRCC and MAD2B-RAN interactions may be dynamic in nature.

In conclusion, we have identified the small GTPase RAN as a novel *bona fide* interactor of the mitotic arrest deficient protein MAD2B. It has been suggested by others that RAN may be involved in cellular transformation through interactions with viral oncogenes [29], [39]. Our data suggest that in t(X;1)(p11;q21)-positive renal cell carcinomas a PRCCTFE3-mediated interference with RAN-dependent MAD2B activity may lead to loss of control over cell cycle progression and, hence, malignant growth.

## Supporting Information

Figure S1 Sub-cellular (co-)localization of MAD2B and RAN. (A) U2OS cells were transiently transfected with MAD2B-GFP and, subsequently, MAD2B was detected in green. Endogenous RAN was detected in red and DAPI staining (blue) was used to mark the position of the nucleus. The overlay of the different signals (Merge; yellow) reveals a near perfect co-localization of the respective proteins. (B) Sub-cellular localization of RAN (red) relative to histone-2B (H2B, green). The overlay of the different signals (Merge) reveals a lack of co-localization. Images were captured using fluorescence microscopy.
(4.44 MB TIF)

Figure S2 Sub-cellular localization of PRCC in U2OS cells. Sub-cellular localization of PRCC (red) relative to histone-2B (H2B, green). The overlay of the different signals (Merge) reveals a lack of co-localization. Images were captured using fluorescence microscopy.
(0.61 MB TIF)
