# Supplementary figures and images for: The Mitotic Arrest Deficient Protein MAD2B Interacts with the Small GTPase RAN throughout the Cell Cycle
Source: PLoS One. 2009 Sep 15;4(9):e7020. doi: 10.1371/journal.pone.0007020 (PMC2737141; doi:10.1371/journal.pone.0007020)

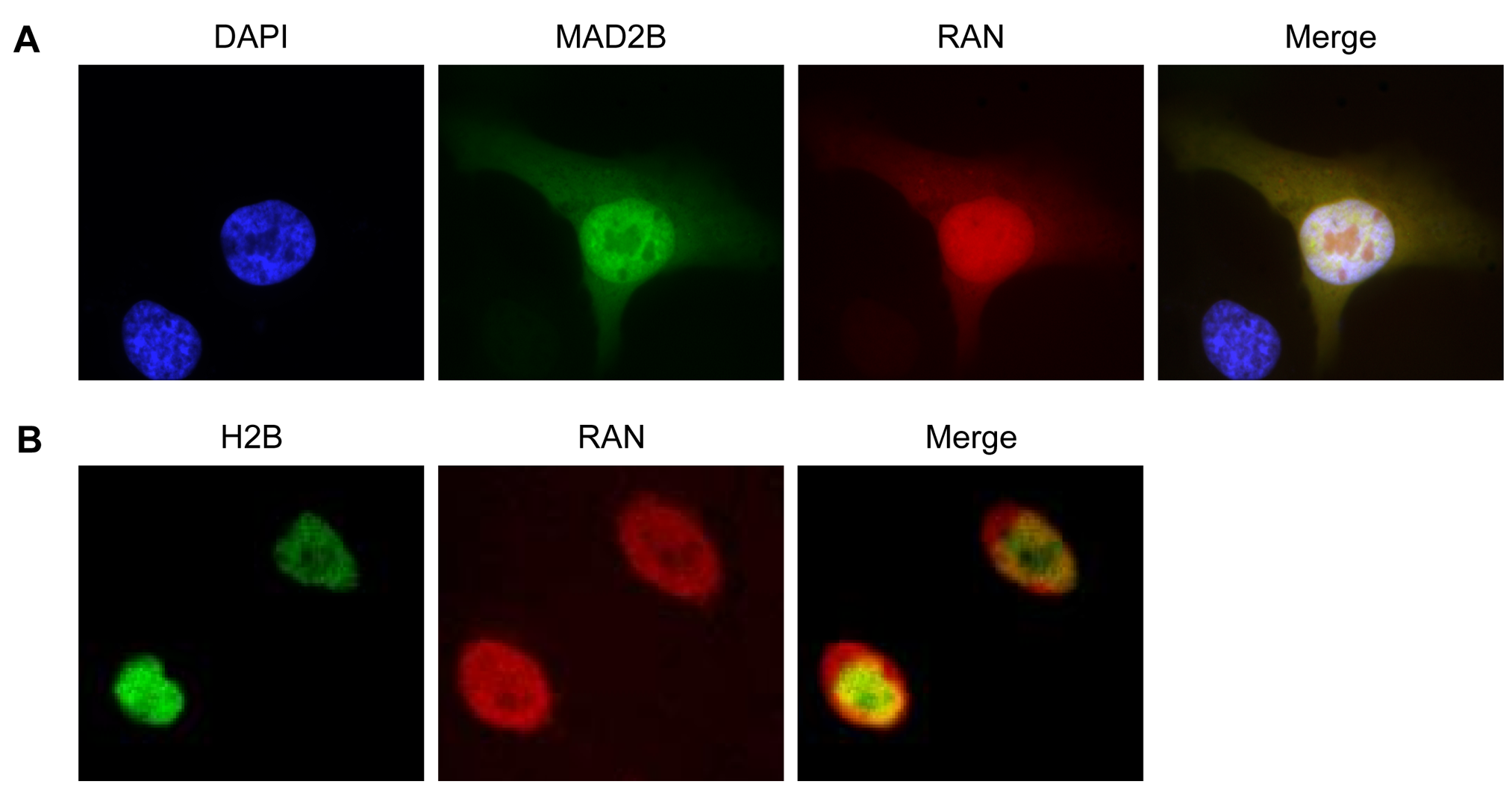

Supplement: Figure S1 — Sub-cellular (co-)localization of MAD2B and RAN. (A) U2OS cells were transiently transfected with MAD2B-GFP and, subsequently, MAD2B was detected in green. Endogenous RAN was detected in red and DAPI staining (blue) was used to mark the position of the nucleus. The overlay of the different signals (Merge; yellow) reveals a near perfect co-localization of the respective proteins. (B) Sub-cellular localization of RAN (red) relative to histone-2B (H2B, green). The overlay of the different signals (Merge) reveals a lack of co-localization. Images were captured using fluorescence microscopy. (4.44 MB TIF) [file pone.0007020.s001.tif]

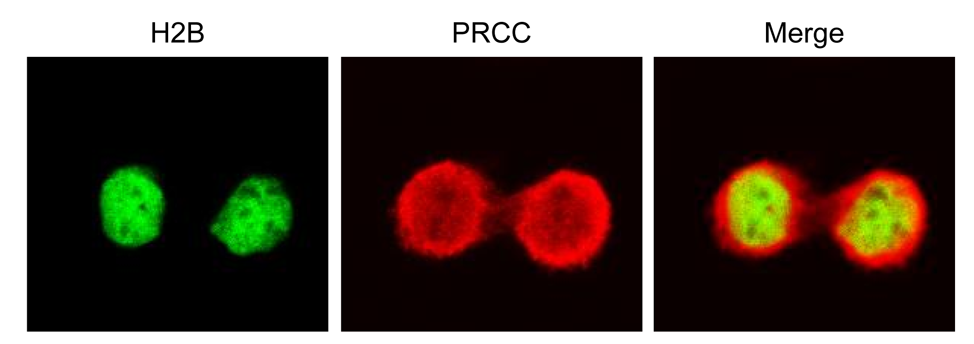

Supplement: Figure S2 — Sub-cellular localization of PRCC in U2OS cells. Sub-cellular localization of PRCC (red) relative to histone-2B (H2B, green). The overlay of the different signals (Merge) reveals a lack of co-localization. Images were captured using fluorescence microscopy. (0.61 MB TIF) [file pone.0007020.s002.tif]
